# Cardiovascular disease and chimeric antigen receptor cellular therapy

**DOI:** 10.3389/fcvm.2022.932347

**Published:** 2022-09-23

**Authors:** Anjali Rao, Andrew Stewart, Mahmoud Eljalby, Praveen Ramakrishnan, Larry D. Anderson, Farrukh T. Awan, Alvin Chandra, Srilakshmi Vallabhaneni, Kathleen Zhang, Vlad G. Zaha

**Affiliations:** ^1^Division of Cardiology, Department of Internal Medicine, UT Southwestern Medical Center, Dallas, TX, United States; ^2^Cardio-Oncology Program, Harold C. Simmons Comprehensive Cancer Center, UT Southwestern Medical Center, Dallas, TX, United States; ^3^Parkland Health and Hospital System, Dallas, TX, United States; ^4^Department of Internal Medicine, UT Southwestern Medical Center, Dallas, TX, United States; ^5^Division of Hematology and Oncology, Department of Internal Medicine, UT Southwestern Medical Center, Dallas, TX, United States; ^6^Myeloma, Waldenstrom's, and Amyloidosis Program, Harold C. Simmons Comprehensive Cancer Center, UT Southwestern Medical Center, Dallas, TX, United States

**Keywords:** chimeric antigen receptor (CAR T), cardio-oncology, immunotherapy, cytokine release syndrome (CRS), cellular therapy, cardiovascular disease

## Abstract

Chimeric antigen receptor T-cell (CAR T) therapy is a revolutionary personalized therapy that has significantly impacted the treatment of patients with hematologic malignancies refractory to other therapies. Cytokine release syndrome (CRS) is a major side effect of CAR T therapy that can occur in 70–90% of patients, with roughly 40% of patients at grade 2 or higher. CRS can cause an intense inflammatory state leading to cardiovascular complications, including troponin elevation, arrhythmias, hemodynamic instability, and depressed left ventricular systolic function. There are currently no standardized guidelines for the management of cardiovascular complications due to CAR T therapy, but systematic practice patterns are emerging. In this review, we contextualize the history and indications of CAR T cell therapy, side effects related to this treatment, strategies to optimize the cardiovascular health prior to CAR T and the management of cardiovascular complications related to CRS. We analyze the existing data and discuss potential future approaches.

## Introduction

The power of the immune system in treating neoplastic diseases has long been recognized in the medical community. However, starting from adoptive cell transfer, the precursor of CAR T, various cardiovascular toxic side effects have also been identified. Herein we review the available data, and propose a strategy for prevention, surveillance and management of cardiovascular toxicity in patients receiving immune cellular therapies.

### Adoptive cell transfer

Adoptively acquired immunity is the process through which active immune tissues are transferred from a donor to a recipient (1–3). Initial studies performed in the 1950's demonstrated in mouse that immune tissue (i.e., spleen or lymph nodes) but not antigens or peripheral cells from a primary transplant tolerant host induced sustained resistance to rejection in a secondary host (1). In a landmark paper published in 1957, E. Donnall Thomas and colleagues demonstrated a sustained response after bone marrow infusion in several patients with bone marrow deficiency following radiation and chemotherapy (3). This led to the first allogeneic bone marrow transplantations in the early 1960s, using bone marrow from twin siblings. With the subsequent development of autologous stem cell transplantation, adoptive cellular therapies have become a mainstay in the treatment of hematologic malignancies (4).

### Modern development of cellular therapies

Following the historic success of bone marrow transplantation, the next phase of adoptive cell transfer came in the 1980s with the emergence of tumor-infiltrating lymphocytes (TIL) (5–8). In this therapy, B- and T-cells isolated from the tumor biopsy are expanded in a laboratory and subsequently infused back into the original host after a dose of chemotherapy (5, 6). TIL were combined with interleukin-2, a key cytokine in the proliferation and differentiation of effector T cells, to enhance their antitumor effects (5, 6).

With the advent of gene-transfer techniques, the potential of peripheral blood T cells was further harnessed through genetic modifications that increase their specificity and augment their function (9, 10). These “first-generation” genetically modified T cells were engineered to express a chimeric antigen receptor (CAR)—composed of an extracellular single-chain variable fragment (scFv) that serves as the targeting moiety, a transmembrane spacer, and intracellular signaling/activation domain(s)—to target surface-exposed tumor-associated antigens (10–12). Over time, CARs evolved to more complex “second-” and “third-generation” CARs that have augmented T cell persistence and proliferation (13–16).

## Chimeric antigen receptor T-cell therapy mechanism and indications

The development of CAR T cell therapy triggered a paradigm shift in cancer immunotherapy, demonstrating remarkable success particularly in CD-19 expressing malignancies, as the first genetically engineered personalized therapy option. This therapeutic option has become a viable and commercially available treatment option for several hematologic malignancies (Table 1). Promising results emerged from the initial CAR T trials of tisagenlecleucel (tisa-cel) and axicabtagene ciloleucel (axi-cel) in 2017 (17). Tisa-cel was the first anti-CD-19 CAR T product approved by the Food and Drug Administration (FDA), for patients up to 25 years of age with relapsed or refractory B-cell precursor acute lymphoblastic leukemia (ALL) in 2017 (17). Axi-cel, an anti-CD-19 targeting CAR T-cell, approval followed soon after in 2017 for patients with relapsed or refractory diffuse large B-cell lymphoma (18). Axi-cel was subsequently also approved for the management of patients with relapsed or refractory follicular lymphoma after 2 prior lines of therapy (19). Since then, the FDA has approved 6 total CAR T therapies for the treatment of hematologic malignancies, including lisocabtagene maraleucel (liso-cel) for relapsed or refractory diffuse large B-cell lymphoma, brexucabtagene autoleucel (brexu-cel) for relapsed or refractory mantle cell lymphoma and relapsed or refractory ALL, and idecabtagene vicleucel (ide-cel) and ciltacabtagene autoleucel (cita-cel) for relapsed and refractory multiple myeloma (18, 20–23) (Table 1). Responses for all these agents average around 60 to 80% with complete remissions achieved in approximately 40 to 60% of the patients (17–19, 21, 22). These results are especially striking given the failure of conventional chemotherapy, including high-dose chemotherapy and stem cell transplantation in this population.

**Table 1:** Summary of current FDA-approved CAR T generic names, trade names, and indications.

**CAR product (generic name)**	**CAR product (trade name)**	**Indication(s)**
Tisagenlecleucel	Kymriah	Acute lymphoblastic leukemia, B-cell lymphoma (17)
Axicabtagene ciloleucel	Yescarta	B-cell lymphoma, follicular lymphoma (18, 19)
Lisocabtagene maraleucel	Breyanzi	B-cell lymphoma (20)
Brexucabtagene autoleucel	Tecartus	Mantle cell lymphoma (21)
Idecabtagene vicleucel	Abecma	Multiple myeloma (22)
Ciltacabtagene autoleucel (cita-cel)	Carvykti	Multiple myeloma (23)

CAR, chimeric antigen receptor.

## Chimeric antigen receptor T-cell therapy induction and administration

The administration of CAR T requires the identification of optimal patients who would generally be considered healthy and fit to undergo this procedure. While there is no established consensus on the optimal patient profile that would be considered suitable, various guidelines suggest utilizing established fitness and morbidity scores to determine eligibility (24–26). After harvesting the peripheral blood product through a routine apheresis procedure, the cells typically require processing and manufacturing which can take up to 4–6 weeks. During this interval, patients frequently require “bridging therapy” to ensure that they do not have rapid and symptomatic disease progression. Following successful manufacturing and receipt of the product, patients undergo lymphodepleting chemotherapy typically with fludarabine and cyclophosphamide over 3 days for up to a week prior to reinfusion of the cells. Patients are subsequently monitored closely for the development of cytokine release syndrome (CRS) and neurotoxicity which can manifest for approximately the first month after reinfusion of cells (24, 25). Because of the risks noted with CRS, patients must enter a risk evaluation and mitigation strategy (REMS) program and stay within 2 h of the CAR T center for the first month and must not drive for 2 months following CAR T.

## Immune cell-related adverse events

Robust systemic release of a high level of cytokines following overwhelming T cell activation as well as specific interactions between the CAR and its target antigen expressed by non-malignant cells are two mechanisms thought to mediate CAR T toxicities (27). One of the most common CAR T cell-related adverse events is CRS. CRS is a multisystem inflammatory response mediated by a surge of cytokines triggered by an infusion of CAR T cells. Among other toxic phenomena, CRS, in particular, affects 37–93% of patients with lymphoma (28), and 77–93% of patients with leukemia (28–31). Clinical manifestations can range from fevers and constitutional symptoms to hypoxia, hypotension, end-organ damage, and even sepsis-like syndrome or death in severe cases (29). CRS is thought to result from widespread simultaneous activation of T-cells and release of cytokines and chemokines (30, 32). CRS has been associated with elevation of interleukin (IL)-6, IL-8, IL-10, IL-15, GM-CSF, interferon (IFN)-g, MCP-1, MIP-1b, ferritin, CRP, and in severe cases soluble IL-2 receptor (28, 33). Management includes supportive care and antipyretics in mild cases, administration of IL-6-receptor antagonists like tocilizumab in moderate CRS or those not responding to supportive care, and corticosteroids like dexamethasone in more severe cases of CRS (34, 35). CRS can occasionally mimic macrophage activation syndrome (MAS) or hemophagocytic lymphohistiocytosis (HLH) in severe cases, which is often treated with anakinra, an IL-1 receptor antagonist, if the above measures are not effective (36–39). Serum inflammatory markers (acute phase reactants) including c-reactive protein (CRP) and ferritin may be followed clinically to help aid in prediction of impending CRS or to monitor response to therapy, though cytokine levels are not often readily available in real time (39).

CRS may contribute to the development of immune cell-associated neurotoxicity syndrome (ICANS), which can manifest along a spectrum from mild delirium with confusion to cerebral edema, seizures, and even death (34, 40). Cardiovascular manifestations of CRS Although the underlying mechanism of ICANS is incompletely understood compared to CRS, studies have also shown a correlation with elevated levels of inflammatory cytokines like IL-6, IFN-γ and TNFα (33, 41, 42). These signals are postulated to cause endothelial damage and activation with disruption of the blood brain barrier and capillary leak. It requires careful monitoring, frequent assessments, and promptly initiated therapy. ICANS has also been associated with sinus bradycardia that is often self-limited without need for intervention but should be monitored closely (43). Other constitutional, hematologic, renal, gastrointestinal, and dermatologic toxicities have also been observed (28, 41, 44–46).

## Cardiovascular complications of cellular imunotherapies

While there has been a consistent trend of improvement in the survival following both autologous and allogeneic hematopoietic cell transplantation bone marrow transplant therapies decade over decade (47, 48), cardiovascular toxicities (49) continue to be frequent complications, along with infections and graft vs. host disease. This has resulted in evolving practice guidelines targeting preventive evaluations pretransplant, monitoring peri-transplant, and surveillance in long term survivors (50, 51). With regard to CAR T therapy, the current information about cardiovascular side effects related to CAR T therapies is limited to a few retrospective studies (Table 2), but concepts established for other adoptive cell transfers likely apply. In particular, with the growing prevalence of cardiovascular disease combined with the increase in available CAR T cell therapies for the treatment of hematologic malignancies, attempting to understand the mechanisms of these complications is essential as this may help guide interventions.

**Table 2:** Summary of pediatric and adult studies investigated cardiovascular complications and CAR T-cell therapy.

**References**	**No. of subjects**	**Oncologic diagnosis**	CAR T*therapy	**Preexisting cardiovascular disease–n (%)**	**Patients with CRS+ [%, (grade)]**	**Adverse cardiovascular events – n (%)**
Fitzgerald et al. (52)^a^	39	Acute lymphoblastic leukemia	CD19-directed T-cells	Not captured	92% (any grade); 46% (3,4)	Vasoplegic shock−13 (36) Cardiomyopathy−1 (2)^c^
Burstein et al. (53)^a^	98	Leukemia/lymphoma	CD19-directed T-cells	Cardiomyopathy−10 (10) Structural disease−6 (6)	24% (≥2)	Shock−24 (24) Cardiac dysfunction−10 (10)^d^
Shalabi et al. (54)^a^	52	Leukemia/lymphoma	CD19-directed T-cells	Not captured	12% (any grade)	Cardiomyopathy−6 (11)^e^ Sinus tachycardia−36 (69)
Alvi et al. (55)^b^	137	Lymphoma, multiple myeloma	axi-cel, tisa-cel	Coronary artery disease−10 (7) Heart failure−5 (4) Atrial fibrillation−18 (13)	59% (any grade); 39% (≥2)	Cardiovascular mortality−6 (4)^f^ Heart failure−6 (4)^e^ Arrhythmia−5 (4)^g^
Ganatra et al. (56)^b^	187	Leukemia/lymphoma	axi-cel, tisa-cel	Coronary artery disease−20 (11)	83% (any grade); 46% (≥2)	Cardiomyopathy−12 (6)^e^ Arrhythmia−13 (7)
Lefebvre et al. (57)^b^	145	Leukemia/lymphoma	axi-cel, tisa-cel	Coronary artery disease−14 (10) Heart failure−12 (8) Atrial fibrillation−4 (3)	72% (any grade)	Heart failure−21 (15)^h^ Atrial fibrillation−11 (7)
Brammer et al. (58)^b^	90	Lymphoma	Axi-cel, tisa-cel, brexu-cel	Coronary artery disease−7 (8) Heart failure−8 (9) Atrial fibrillation−10 (11)	49% (≥2)	Arrhythmia−11 (12)^i^ Myocarditis−2 (2) Heart failure−1 (1)^h^
Steiner et al. (59)^b^	165	Lymphoma	axi-cel, tisa-cel	Coronary artery disease−15 (9) Heart failure−14 (8)	14% (≥3)	Arrhythmia−15 (9)^j^ Heart failure−3 (2)^h^ Myocardial infarction−3 (2)^k^

*CAR T, chimeric antigen receptor T-cell + CRS, cytokine release syndrome.

Study specific parameters: ^*a*^ Pediatric population; ^*b*^ Adult population; ^*c*^ Cardiomyopathy, defined as decreased left ventricular systolic function requiring milrinone; ^*d*^ Cardiac dysfunction, defined as either an echocardiographic decrease of ≥10% in ejection fraction or ≥5% in shortening fraction from normal baseline ejection fraction > 55% or shortening fraction > 28%; ^*e*^ Cardiac dysfunction, defined as either a >10% absolute decrease in LVEF compared with baseline or new-onset LV systolic dysfunction (LVEF < 50%); ^*f*^ Cardiovascular mortality, defined as a combination of death due to heart failure, cardiogenic shock, cardiac arrest, or an arrhythmia; ^*g*^ Arrhythmia, defined as new-onset supraventricular tachycardia, atrial fibrillation, or atrial flutter requiring intervention; ^*h*^ Heart failure, defined as clinical signs of heart failure on physical examination, laboratory or imaging or radiographic findings of heart failure (B-type natriuretic peptide or N-terminal pro–B-type natriuretic peptide, Kerley B-lines or pulmonary edema, pleural effusion, decreased left ventricular ejection fraction, and initiation of new treatment for heart failure (pharmacological therapies such as diuretic agents and/or mechanical support); ^*i*^Arrhythmia, defined as atrial fibrillation, ventricular tachycardia; ^*j*^ Arrhythmia, defined as non-sustained ventricular tachycardia, atrial fibrillation; ^*k*^ Myocardial infarction, defined as angina or anginal equivalent symptoms with cardiac enzyme elevation, with or without EKG/echocardiographic changes.

PubMed search performed using the following terms: Chimeric antigen receptor; cardiovascular; cytokine release syndrome.

The impact of CAR T cell therapy on the cardiovascular system manifests as hemodynamic compromise, myocardial injury/dysfunction, and/or cardiac arrhythmias (60, 61). There is also the potential for pericardial complications, such as in a case report (62) describing a patient with high-grade lymphoma who developed a pericardial effusion and tamponade with cardiogenic shock after CAR T therapy. Higher-grade CRS appears to be linked to adverse cardiovascular events of all types. This is likely driven by the release of inflammatory cytokines into the bloodstream with CAR T therapy, particularly the secretion of interleukin-6 (IL-6). This cytokine is a mediator of systemic inflammation, leading to hemodynamic compromise and even circulatory collapse in CRS. Of the studies published so far in patients treated with CAR T cell therapy, cardiovascular monitoring was performed in 3 pediatric studies and 5 adult studies (52–59). All studies in adult populations were retrospective, single-center observational cohort studies. Across all studies, cardiovascular complications have been inconsistently monitored. In children, transient and reversible hypotension in the setting of high-grade CRS was more commonly noted. In studies that monitored for cardiovascular complications in adults, the most frequently observed were cardiac arrhythmias and heart failure, albeit with relatively low event rates overall. Interestingly, preexisting cardiovascular disease (including heart failure) has not been shown to be reliably associated with the development of cardiovascular complications after CAR T cell therapy in one cohort study (57). In contrast, in another cohort study (55), troponin elevation was notably associated with cardiovascular adverse events in patients undergoing CAR T cell therapy. The patients with troponin elevation in this study were older and had more traditional cardiovascular risk factors. In both these cohort studies cardiovascular complications occurred with increased frequency at higher grades of CRS (2 or greater). As such, additional studies in larger cohorts are needed to establish risk factors, biomarker elevation patterns, imaging findings, event rates, and outcomes after CAR T cell therapy.

### CRS monitoring and grading

Most patients undergoing CAR T can be managed on the regular cell therapy hospital floor with only a minority requiring ICU care, but close monitoring and specialty care is. due to rapid onset of CRS, it is recommended that this therapy is given at a specialized center with CAR T experience and credentialling.

Grading of CRS is now done per the American Society of Transplantation and Cellular Therapy (ASTCT) consensus guidelines (Table 3) (34).

**Table 3:** American Society of Blood and Marrow Transplantation (ASBMT) consensus grading of cytokine release syndrome (CRS) severity (34).

**Cytokine release syndrome parameter**	**Grade 1**	**Grade 2**	**Grade 3**	**Grade 4**
Fever	Temperature ≥38°C	Temperature ≥38°C	Temperature ≥38°C	Temperature ≥38°C
With				
Hypotension	None	Not requiring vasopressors	Requiring one vasopressor with or without vasopressin	Requiring multiple vasopressors (excluding vasopressin)
And/or*				
Hypoxia	None	Requiring low-flow nasal cannula or blow-by	Requiring high-flow nasal cannula, facemask, nonrebreather mask, or Venturi mask	Requiring positive pressure (e.g., CPAP, BiPAP, intubation, and mechanical ventilation)

CPAP, Continuous Positive Airway Pressure; BiPAP, Bilevel Positive Airway Pressure.

*CRS grade is determined by the more severe event: hypotension or hypoxia not attributable to any other cause.

Fever is defined as temperature≥38°C not attributable to any other cause. In patients who have CRS then receive antipyretic or anti-cytokine therapy such as tocilizumab or steroids, fever is no longer required to grade subsequent CRS severity.

Low-flow nasal cannula is defined as oxygen delivered at ≤ 6 L/min. Lowflow also includes blow-by oxygen delivery, sometimes used in pediatrics. High-flow nasal cannula is defined as oxygen delivered at>6 L/min.

### CRS management

Rates of CRS and median time to onset vary depending on the particular CAR T product and disease burden. For example, in the KarMMa study of ide-cel for relapsed/refractory multiple myeloma (22), CRS was seen in 84% of patients, but most cases were only grade 1 or 2, with only 5% of patients developing grade 3–5. Median time to onset of CRS in the KarMMa study was 1 day (range 1–12 days) with a median duration of 5 days (range 1–63).

Management of CRS required tocilizumab in 52% patients, but only 15% required glucocorticoids (22, 63). On the other hand, in the Zuma-1 study of axi-cel for relapsed/refractory large B-cell lymphomas, CRS was a nearly universal side effect, with 93% of patients experiencing any grade CRS and 11% with grade 3 or higher, and hypotension was seen in 63%, tachycardia in 40%, and hypoxia in 34% (64). The median time to onset of CRS was 2 days (range 1–12) with a median duration of 8 days (65). All patients had resolution of their CRS, except for one patient who died from complications of HLH, and another patient who died of cardiac arrest with ongoing CRS. Tocilizumab was given in 43% and corticosteroids were required in 27% of Zuma-1 patients; however, more recently the FDA has issued a new label change for axi-cel allowing the prophylactic use of 3 days of corticosteroids based on a study showing much less severe CRS and ICANS without impairment of lymphoma response rates (66). The decision regarding inpatient vs. outpatient care and aggressive early therapy vs. minimal therapy for CRS is not only made based on the track record of the particular CAR T product but also based on risk factors such as age, frailty, and tumor burden, as higher tumor burden consistently correlates with increased incidence and severity of CRS (67).

## Surveillance for cardiovascular toxicity

At our institution, cardiovascular (CV) surveillance for CAR-T therapy begins with CV risk stratification prior to infusion. Patients with CV comorbidities (especially heart failure, coronary artery disease, arrhythmias) or new/worsening CV symptoms (i.e., chest pain, dyspnea on exertion, lower extremity edema) represent a high CV risk group. Older age and prior cardiotoxic cancer therapy (i.e., anthracyclines, chest radiation) may also raise the risk of CV toxicity after treatment (68). In these high CV risk patients, standard baseline testing should include a 12-lead electrocardiogram, cardiac biomarkers (troponin, NT-proBNP), and transthoracic echocardiography. In some cases, cardiac MRI may clarify features of cardiac structure and/or function that would guide optimization of CV therapy. Cardioprotective therapies such as beta-blockers and renin-angiotensin-aldosterone system blockers, diuretics, and/or antiarrhythmics should be utilized as clinically indicated. In addition, any patient with the above cardiovascular comorbidities, and whose baseline electrocardiogram or transthoracic echocardiogram is abnormal, should be considered for cardio-oncology referral pre-CAR T therapy.

**Figure 1:**
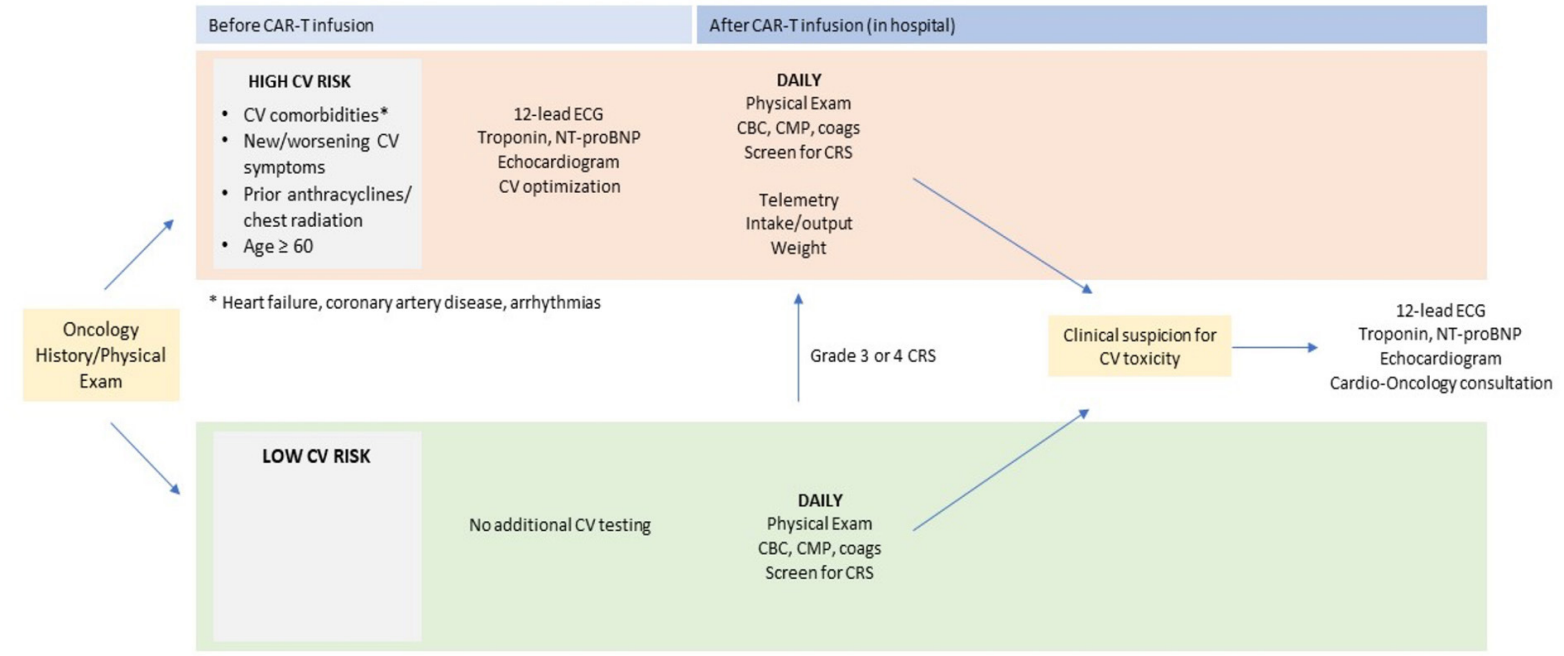
Proposed pre- and post-CAR T cardiac screening. CAR, chimeric antigen receptor; ECG, electrocardiography; NT-proBNP, N-terminal pro–B-type natriuretic peptide. *arrhythmias, coronary artery disease, heart failure.

Inpatient monitoring after CAR-T infusion is strongly recommended for patients with increased baseline CV risk. Figure 1 shows our institutional algorithm for surveillance and monitoring in this population. Standard monitoring protocols after CAR-T infusion include daily blood counts and metabolic profiling, physical examination, and screening for CRS (69). Patients at high baseline CV risk should additionally be monitored on telemetry with close monitoring of oral and intravenous fluid input, urine output, and daily body weight measurement. Given the observed association between CRS and CV events after CAR-T (55, 57), all patients with grade 3 or 4 CRS should also be placed on these CV monitoring protocols.

The utility of routine cardiac biomarker testing for detection of CV toxicity after CAR-T is uncertain. Where there is clinical suspicion for a CV event after CAR-T infusion based on symptoms or monitoring, initial evaluation should include cardiac biomarkers (troponin, NT-proBNP), 12-lead electrocardiogram (ECG), and transthoracic echocardiography (TTE). Cardio-oncology consultation should be obtained, if available, to direct further diagnostic evaluation and management.

Data are limited regarding the optimal surveillance and testing protocol for patients undergoing CAR T cell therapy. Current standards of practice have been previously published by Hayden et al. (26), Ghosh et al. (60), and Totzeck et al. (70), with similar approaches to our institution and each other with regard to screening and surveillance while on CAR T therapy. Ghosh et al. propose that all patients undergo baseline cardiac magnetic resonance imaging (CMR) with follow-up CMR in patients with abnormal biomarkers, ECG, and/or TTE. We generally agree with these publications on the initial evaluation of patients after a cardiovascular event with CAR-T infusion, including cardiac blood biomarkers (troponin, NT-proBNP), ECG, and TTE, with judicious use of CMR in appropriate cases. By contrast, there is some variability in the post-CAR T surveillance and monitoring approaches proposed by the other consensus approaches. For example, Ghosh et al. recommend for all patients to follow-up with cardio-oncology 3 months after CAR T cell therapy, whereas the other two consensus recommendations propose a 7-day follow-up visit. We propose a patient-specific approach depending on the type of cardiovascular event that patient experienced. The utility of monitoring for late effects (i.e., at 3 months post CAR-T and beyond) and the potential for long-term CV consequences of CAR-T itself stand out as areas for future study.

## Future directions

Current targets of CAR T are malignant immune cells, but new targets continue to develop. There has been an expanding focus on targeting solid tumors, and overall, nearly 600 clinical trials are underway (71–73). Multiple new endeavors are focusing on solid tumor surface antigens such as carcinoembryonic antigen (CEA), ganglioside GD2 subtype, mesothelin, interleukin-13 receptor α (IL-13Rα), human epidermal growth factor receptor 2 (HER2), fibroblast activation protein (FAP), and L1 cell adhesion molecule (L1CAM) (16, 74–79).

Multiple trials are currently ongoing evaluating various CAR T products in different disease entities including allogeneic products utilizing various T-cell and NK-cell engineering and manufacturing procedures. Moreover, the well-documented side effects of CAR T–most notably, CRS–have spurred the recent discussion surrounding CAR NK-cell therapy, a potential avenue to mitigatehe systemic immune effects (73). CAR T has been shown to effectively target and remove activated cardiac fibroblasts in mice, suggesting potential applications to address myocardial scar and fibrosis (80, 81). At the same time, early signals have raised concerns about the unique dangers of systemic immune effects in patients with preceding cardiovascular diseases or cardiovascular risks, with limited information about cardiotoxicity available from the initial CAR T trials. Clinical practice guidelines are emerging to address immune cell-related adverse events (82). Next steps also include validated risk prediction tools for cardiovascular complications after CAR-T, elucidate mechanisms of these immune-mediated complications, development of preventative therapies by integrating timelines of cardiac blood biomarkers and immunophenotyping in this population.

## Conclusions

The rapid development of immunocellular personalized therapeutic modalities is creating unprecedented opportunities for treatment of cancers. To optimize the cardiovascular outcomes in patients treated with CAR T several lessons learned from other anticancer therapies and from early CAR T studies may be beneficial. While early studies have established the specific indications for these therapies, cardiovascular risk profiles will need to be defined further during their real-life application. The awareness of interactions between the cardiovascular risks, underlying cardiovascular problems and the cytokine release syndrome is prompting the definition of systematic assessments before and during CAR T therapy. Yet unknown potential latent effects, such as vascular inflammation seen after other immunotherapeutic interventions (i.e., immune checkpoint inhibitor therapies) will need to be taken in consideration for long-term cardiovascular surveillance. Inclusion of cardiovascular endpoints in trials, as well as broad collaborative, prospective clinical registries have the potential to provide new information about these risks. And not the least, the further investigation of such observations in targeted research studies has the potential to refine this technology and expand its safe applicability.

## Author contributions

AR and VZ organized the outline and the components of the manuscript, as well. AR, AS, and ME performed an extensive literature search of cardiovascular disease and chimeric antigen receptor T cell therapy. PR, LA, and FA wrote individual sections on CAR T therapy and provided feedback on the manuscript. AC, SV, and KZ created the figure and wrote the section on surveillance and monitoring. All authors contributed to the writing efforts and the editing of this manuscript.

## Funding

VZ received support from the Cancer Prevention Research Institute of Texas (RP180404).

## Conflict of interest

Author FA has provided consultancy to: Genentech, Astrazeneca, Abbvie, Janssen, Pharmacyclics, Gilead sciences, Kite pharma, Celgene, Karyopharm, MEI Pharma, Verastem, Incyte, Beigene, Johnson and Johnson, Dava Oncology, BMS, Merck, Cardinal Health, ADCT therapeutics, and Epizyme. The remaining authors declare that the research was conducted in the absence of any commercial or financial relationships that could be construed as a potential conflict of interest.

## Publisher's note

All claims expressed in this article are solely those of the authors and do not necessarily represent those of their affiliated organizations, or those of the publisher, the editors and the reviewers. Any product that may be evaluated in this article, or claim that may be made by its manufacturer, is not guaranteed or endorsed by the publisher.

## Abbreviations

ALL: acute lymphocytic leukemia
CAD: coronary artery disease
CAR T-cell: chimeric antigen receptor T-cell
CAR NK-cell: chimeric antigen receptor natural killer cell
CEA: carcinoembryonic antigen
CHF: congestive heart failure
CMR: cardiac magnetic resonance
CRS: cytokine release syndrome
CV: cardiovascular
CVD: cardiovascular disease
ECG: electrocardiogram
FAP: fibroblast activation protein
FDA: Food and Drug Administration
GD2: disialoganglioside 2
HER2: human epidermal growth factor receptor 2
HLH: hemophagocytic lymphohystiocytosis
ICANS: immune cell-associated neurotoxicity syndrome
IFN-γ: interferon-gamma
IL: interleukin
L1CAM: L1 cell adhesion molecule
MCP-1: monocyte chemoattractant protein-1
MI: myocardial infarction
MIP-1β: macrophage inflammatory protein-1 beta
REMS: risk evaluation and mitigation strategy
TIL: tumor-infiltrating lymphocytes
TNFα: tumor necrosis factor alpha
TTE: transthoracic echocardiogram.
